# Exploring the Impact of Developmental Clearance Saturation on Propylene Glycol Exposure in Adults and Term Neonates Using Physiologically Based Pharmacokinetic Modeling

**DOI:** 10.1002/jcph.6150

**Published:** 2024-10-15

**Authors:** Olusola Olafuyi, Robin Michelet, Michael Garle, Karel Allegaert

**Affiliations:** ^1^ Division of Physiology, Pharmacology and Neuroscience, School of Life Sciences University of Nottingham Nottingham UK; ^2^ Department of Clinical Pharmacy and Biochemistry Institute of Pharmacy Freie Universität Berlin Berlin Germany; ^3^ qharmetra LLC Berlin Germany; ^4^ Department of Development and Regeneration KU Leuven Leuven Belgium; ^5^ Department of Pharmaceutical and Pharmacological Sciences KU Leuven Leuven Belgium; ^6^ Department of Hospital Pharmacy Erasmus Medical Center Rotterdam The Netherlands

**Keywords:** excipient, neonates, ontogeny, physiologically based pharmacokinetics, propylene glycol

## Abstract

Propylene glycol (PG) is a pharmaceutical excipient which is generally regarded as safe (GRAS), though clinical toxicity has been reported. PG toxicity has been attributed to accumulation due to saturation of the alcohol dehydrogenase (ADH)‐mediated clearance pathway. This study aims to explore the impact of the saturation of ADH‐mediated PG metabolism on its developmental clearance in adults and neonates and assess the impact of a range of doses on PG clearance saturation and toxicity. Physiologically based pharmacokinetic (PBPK) models for PG in adults and term neonates were developed using maximum velocity (V_max_) and Michaelis–Menten's constant (K_m_) of ADH‐mediated metabolism determined in vitro in human liver cytosol, published physicochemical, drug‐related and ADH ontogeny parameters. The models were validated and used to determine the impact of dosing regimen on PG clearance saturation and toxicity in adults and neonates. The V_max_ and K_m_ of PG in human liver cytosol were 1.57 nmol/min/mg protein and 25.1 mM, respectively. The PG PBPK model adequately described PG PK profiles in adults and neonates. The PG dosing regimens associated with saturation and toxicity were dependent on both dose amount and cumulative in standard dosing frequencies. Doses resulting in saturation were higher than those associated with clinically observed toxicity. In individuals without impaired clearance or when PG exposure is through formulations that contain excipients with possible interaction with PG, a total daily dose of 100‐200 mg/kg/day in adults and 25‐50 mg/kg/day in neonates is unlikely to result in toxic PG levels or PG clearance saturation.

## Introduction

Besides active pharmaceutical substance(s), formulations of drugs usually contain solvents and excipients. These excipients are added to ensure solubility and stability of the drug over a given shelf life, under variable external conditions or to improve palatability. Propylene glycol (PG) is one of the alcohols commonly considered as excipient. Although PG has the generally regarded as safe (GRAS) status, concentration‐related toxicity due to accumulation has been reported in adults and children. This toxicity involves increase in osmolar gap, anion gap, lactic acidosis, hepatic dysfunction, or kidney injury and central nervous system symptoms.^1^, ^2^

In adults, 45% of PG is eliminated through the renal route and 55% is metabolized in the liver by the cytosolic enzyme, alcohol dehydrogenase (ADH) to lactate and pyruvate.^3^, ^4^, ^5^, ^6^, ^7^ ADH activity is reduced in neonates, reaching adult levels from about 5 years of age onward.^8^ Furthermore, ADH activity is well known for its saturability, resulting in a shift from first‐ to zero‐order kinetics. Due to immature renal function, renal PG clearance is likely further decreased in neonates compared to adults.^9^

The association between dose, plasma concentration, and toxicity of PG is inconsistent in the published literature, perhaps due to potential patient‐related, medication‐related, and dosing strategy‐related confounding factors. For example, reversible metabolic abnormalities up to clinical deterioration were reported in some adults who receive PG‐containing benzodiazepines in a prospective study reported by Wilson et al (2005).^4^ These toxic side effects were attributed to plasma PG concentration ranging from 58 to 144 mg/dL. Similarly, in 8 out of 128 critically ill adults receiving PG‐containing lorazepam, clinical signs of renal dysfunction, hyperosmolality, and metabolic acidosis were observed with the administration of mean cumulative PG doses of 0.51 to 1.19 g/kg/day. However, other studies showed that with short term infusion of PG of up to 1.6 g/kg/day^10^ or 1.0 g/kg/day,^11^ there were no signs of renal dysfunction or failure observed in adults. Similarly, Yahwek et al (2008) demonstrated that no signs of renal dysfunction were observed in adults when a low dose PG (0.078 g/kg/day) was administered as continuous infusion for 3 weeks. In the same study, however, a 0.62 g/kg/day continuous infusion of PG (equivalent to about 123 mg of lorazepam per day) resulted in increased serum creatinine from about 6 days following the start of the infusion.^12^

In neonates, PG toxicity in very low birth weight (<1500 g) infants has been reported during exposure of up to 3000 mg/24 h for at least 5 consecutive days.^13^, ^14^, ^15^ Such an exposure was due to high PG concentrations as co‐solvent in parenteral nutrition formulations in the 1980s. The toxicity exhibited both clinical (seizures) and biochemical symptoms (hyperosmolarity, lactic acidosis, plasma creatinine, and raised bilirubin). The elimination half‐life was estimated to be 10‐31 h, compared to 2‐5 h in adults.^13^, ^14^, ^15^ More recently in 2011, the US Food and Drug Administration (FDA) notified healthcare professionals of serious health problems that had been reported in premature babies receiving Kaletra (lopinavir/ritonavir) oral solution.^16^ This is an antiviral medication used in combination with other antiretroviral drugs for the treatment of HIV‐1 infection. However, this oral solution contains both relevant amounts of ethanol (356.3 mg/mL) and PG (152.7 mg/mL). Based on the reported side effects, the FDA warned that premature babies may be at risk for serious health problems due to their decreased ability to eliminate PG, possibly leading to adverse events such as serious and severe heart, kidney, or breathing problems.^16^

In the European Medicines Agency (EMA) report on PG, it was stated that clinical data showed that in children from the age of 5 years and adult patients, up to 500 mg/kg/day of PG could generally be considered safe. In the absence of compelling data, this safety threshold is decreased to 50 mg/kg/day in children less than 5 years old, and even to 1 mg/kg/day in (pre)term neonates.^2^ These EMA recommendations are based on clinical observations of PG exposure under less well‐defined circumstances. For example, the presence of disease or coadministration of drugs containing excipients (like ethanol) which may interact with PG, and the dosing duration or strategy may be confounding factors while examining the association of PG exposure to toxicity and adverse clinical outcomes.^9^

Since this statement, physiologically based pharmacokinetic (PBPK) tools emerged as a structured approach with improving confidence in their use, up to regulatory use and approval.^17^, ^18^ PBPK modeling is a knowledge‐driven technique that applies mathematical models for mechanistic integration of pharmacological principles, assumptions, and data along the drug development process. It thereby integrates different types of information, and PBPK explicitly discriminates between physiological properties of the population (system) and compound specific properties. These PBPK tools are also increasingly developed and used for neonates.^19^, ^20^, ^21^ Besides its use to explore the pharmacokinetics of active compounds, these tools can also be used to explore and better understand the pharmacokinetics of excipients, like PG as demonstrated in the use of PG modeling to assess the concentration–time profiles of ethanol in different organs under fed and fasted conditions.^22^ The aim of this study was therefore to use PBPK modeling to predict the developmental saturation of PG clearance, and to determine the clinical dose associated with saturable clearance and PG toxicity in neonates and adults, applying an increase in osmolar gap as the first signal of toxicity.

## Methods

### In Vitro Determination of Enzyme Kinetic Parameters

#### Chemicals and Human Liver Cytosol

The chemicals sourced for this study were of the highest available purity. They include: PG purchased from Sigma‐Aldrich; nicotinamide adenosine dinucleotide (NAD) and nicotinamide adenosine dinucleotide hydrogen (NADH) purchased from Sigma‐Aldrich; potassium dihydrogen phosphate (KH_2_PO_4_) and dipotassium phosphate (K_2_HPO_4_) purchased from Fisher Scientific; and disulfiram purchased from LGC Dr Ehrenstorfer. Pooled human liver cytosol (HLC) was purchased from Sigma‐Aldrich.

#### Determination of PG Metabolism Kinetic Parameters

The kinetics of PG metabolism was measured using the NADH detection method for alcohols.^23^ As NADH is the product of both PG metabolism via the ADH enzyme pathway and its metabolite—lactaldehyde metabolism via the aldehyde dehydrogenase (ALDH) enzyme pathway, NADH formed via the lactaldehyde pathway was blocked with disulfiram^24^, ^25^ which is an inhibitor of ALDH enzyme.

The NADH standard curve, optimal cytosolic protein concentration, and incubation time with linear metabolism of PG were determined and detailed in the Supplemental Information.

Once linearity of PG metabolism was confirmed, preincubation of 0 mM, 1 mM, 3 mM, 10 mM, 30 mM, 100 mM, 300 mM, and 1 M of PG were performed in triplicates in 96‐well. Each preincubation mixture was a total volume of 200 µL, and in addition to PG, consisted of 0.1 M phosphate buffer (KH_2_PO_4_/K_2_HPO_4_, pH 7.4), 1 mM NAD+ and 0.5 mg/mL HLC, and 20 µM disulfiram which served as ALDH inhibitor. To initiate incubation and the metabolic reaction, the 96‐well plate was incubated at 37°C. The absorbance of NADH was taken at a wavelength of 340 nm using the Spectramax M2e multi‐detection microplate reader at start of incubation and at 20 min.

#### In Vitro Data Analysis

The NADH standard curve was used to convert absorbance values to the concentration of NADH produced. The resultant NADH concentrations were then converted to amount in moles of NADH formed per minute per mg of cytosolic protein (nmole/min/mg of protein).

The rates of NADH formation were plotted against the concentrations of PG and fitted to a Michael–Menten equation on GraphPad Prism (V9) to estimate the maximum rate of NADH formation (V_max_) and the concentration of PG at which half of the maximum rate of NADH formation is achieved (K_m_). For every mole disappearance of PG, there is a one mole production of NADH (Figure S1), therefore, the rate of NADH formation was said to be equivalent to the rate of PG disappearance.

The enzymatic activity resulting in the formation of NADH in the reaction was attributed solely to alcohol dehydrogenation by ADH because ALDH enzymatic activity which has a minute contribution to NADH production, was inhibited using disulfiram.^24^, ^25^ The contributions of individual ADH isoforms were not determined, hereby accounting for all ADH‐mediated metabolic reactions in the liver cytosol.

### PG Physiologically Based Pharmacokinetic (PBPK) Model Development

The PBPK models used for predicting the pharmacokinetics of PG in adults and term neonates were developed in Simcyp (Simcyp Ltd., a Certara company, Sheffield, UK, Version 20). The Simcyp simulator has built‐in virtual pediatric and adult populations which have been previously validated.^26^, ^27^, ^28^ Published physiological and biochemical parameters of pediatrics and adults populations^29^, ^30^ were used to develop the respective virtual populations in the Simcyp simulator and the interindividual variabilities in those parameters as expected in the real population were incorporated in the virtual human models.^31^ To account for the impact of age‐related changes in physiology and biochemistry on the PK of PG in neonates, the built‐in neonatal age group of the virtual pediatric population within the simulator was used for simulations of PG PK. The simulator's pediatric population incorporates the developmental changes in body weight, body surface area, and body composition expected in neonates.^27^, ^29^, ^32^, ^33^ Physicochemical^34^ and other compound‐related data of PG were used to develop the PG model in adult and neonates (Table 1). Unless otherwise stated, population sizes used during simulations included 200 virtual subjects. Figure 1 depicts the steps taken in developing the PG PBPK model following a learn–predict–confirm paradigm^35^ and simulating the saturable clearance of PG. Details of the PG model structure and development process in adults and neonates are reported in Figure S3 and Section S1, respectively.

**Table 1:** Parameters Used to Develop PG PBPK Model in Adults and Pediatrics

Parameter	Optimized Adult Model	Optimized Pediatric Model
**Compound type**	Neutral	
**Molecular weight (g/mol)**	76.06^43^	
**log P**	0.92^43^	
**fu_p_**	0.99^6^	
**B:P**	1.2^a^	
**Absorption model**	First order	
**fa**	0.99^a^	
**k_a_ (per h)**	7.56^a^	
**Distribution model**	Full	
**Vss (L/kg)**	0.80^c,^ ^6^	0.40^38^
**Kp scalar**	1.0	0.44^b^
**CL_R_ L/h**	3.3^d, # 6^	
**Tissue scalar _liver_**	11^b^	
**F_ADH activity, neonate_ (%)**	18 ^b,e^	

B:P, blood‐to‐plasma ratio; CL_R,_: renal clearance; fa, fraction of dose absorbed; F_ADH activity,neonate_; fraction of adult ADH activity in neonates; fu_p,_ unbound fraction in plasma; ka, absorption rate constant; Kp scalar, scalar applied to all predicted tissue partition values; log P, the logarithm of the n‐octanol:buffer partition coefficient; pKa, dissociation constant; tissue scalar, applied to compensate for the differences in intrinsic activity per unit enzyme expressed in HLC relative to the whole organ; Vss, steady state volume of distribution.

a Simcyp mechanistic prediction based on physicochemical properties of compound.

b Empirically optimized using weighted least square (WLS) objective function and the Nelder–Mead minimization method.

c Calculated average volume of distribution reported in literature.^6^

d Calculated based on a 45% estimated contribution to total CL (0.1 L/kg) as reported in Yu et al (1985).^44^

e Estimation based on a calculated average expression of 13.7% in less than 1 month old neonates in Pikkarainen et al (1967).^8^

# Estimation in pediatric was based on pediatric renal clearance reported in De Cock et al (2013).^38^

**Figure 1:**
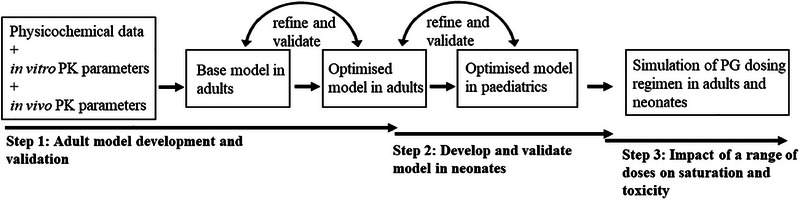
The steps involved in the development and validation of PG PBPK model in adults and neonates.

#### PG Model Development in Adults

As PG undergoes rapid gastrointestinal absorption, a first‐order absorption model was used to simulate the absorption kinetics of oral PG administration. In addition, the “full PBPK distribution” model within the simulator was utilized to predict the distribution kinetics of PG. The “full PBPK distribution model” which is based on tissue: plasma partition coefficients (K_p_) the model proposed by Poulin and Theil^36^ and modified by Berezhkovskiy^37^ (method 1 in Simcyp) were used to simulate the volume of distribution at steady state (V_ss_) in the virtual adults (Table 1). The structure of full PBPK model used is shown in Figure S5. To simulate the metabolism kinetics of PG, the V_max_ and K_m_ of PG from HLC which was determined using the methodology described in the main text were utilized. More precisely, the Simcyp user cyst 1 option for cytosolic enzymes was changed to ADH in the enzyme kinetic screen on the Simcyp software, followed by inputting the V_max_ and K_m_ of PG in HLC. To recover the full contribution of hepatic clearance to PG elimination, a tissue scalar was parameter estimated using weighted least square (WLS) and Nelder–Mead minimization approach within Simcyp. The tissue scalar is an inbuilt function which is used to compensate for the differences in intrinsic activity of enzyme expressed in HLC relative to the whole organ. As the liver ADH expression is the main driver of ADH activity systemically,^23^, ^38^ a tissue scalar was applied only to the liver. The renal excretion of PG was incorporated in the model which accounted for 45% of its published total systemic clearance.^39^

#### PG Model Development in Term Neonates

The same “full PBPK distribution model” used in adults was implemented for predictions in the pediatric populations. However, to recover the observed distribution profile of PG in pediatrics, a scalar was applied to the predicted K_p_ for each tissue. The scalar applied was estimated using the WLS objective function and the Nelder–Mead minimization method within the simulator (Table 1). The estimation was based on one of the plasma concentration datasets in neonates which were available for the model validation in this current study.

To dynamically account for ADH activity with increasing pediatric age, Equation (1), was applied. This accounted for changes in ADH activity in children relative to adults ADH activity

(1) $$\mathrm{Fractional}\;\mathrm{change}\;\mathrm{with}\;\mathrm{age}=F_{\mathrm{birth}}+\frac{(F_{\max}-F_{\mathrm{birth}})\times \mathrm{Ag}e^{n}}{{\mathrm{Age}}_{50}^{n}+\mathrm{Ag}e^{n}}$$

where F_max_ (maximal fraction of expression at a given age) = 1; F_birth_ (fraction at birth) = 0.18; Age_50_ (age to half maximum expression) = 0.9; n (exponent) = 1.4; and Age is age in years.

ADH neonatal activity data collected directly from Pikkarainen et al's (1967) study,^8^ suggests that in full‐term neonates, ADH activity is about 13% of adult. Bhatt et al (2017)^7^ reported ADH abundance of isoforms when considered in combination, the total ADH abundance is about 11% of adult levels suggesting that activity of ADH in neonates likely correlates with the abundance levels (Table S1). The fractional ADH enzymatic activity (F_birth,ADH_) used in the model was optimized using the WLS and Nelder–Mead minimization approach from 13% to 18% (Table 1) to recover the fraction metabolized in neonates as reported in DeCock et al.^39^ The final parameterized value of 18% was congruent with reports by Marek et al (2014)^40^ and Chung et al (2024)^41^ that the activity of ADH in neonates is between 10% and 20% of adult activity. As in adults, the potential ADH expression in other neonatal organs were not accounted for since the ADH isoforms expressed in this organs (class V and II)^42^ have insignificant contribution to ADH metabolic activity.^23^ The renal function of neonates, that is, the glomerular filtration rate (GFR) was determined using Equation (2).

(2) $$\mathrm{GFR}(\mathrm{mL}/\min)=a_{0}+(a_{1}\times \mathrm{BSA})+(a_{2}\times \mathrm{BS}A^{2})$$

where $a_{0}$, $a_{1},$ and $a_{2}$ are −19.8, 89.04, and −7.16, respectively, and were estimated based on renal clearance of PG reported in De Cock et al (2014).^43^

### Validation of PG Models in Adults and Neonates Using Clinically Determined Plasma Concentrations of PG

The concentration–time profiles and pharmacokinetic parameters of PG in adults as reported in two published clinical studies were used to validate the predicted plasma concentration profile and pharmacokinetic parameters predicted by the model. A summary of the clinical studies used for the validation of PG model in adults is shown in Section S2 (Table S3). The reported concentration–time profiles from the clinical studies were digitally retrieved using the WebPlotDigitizer v3.10.^44^

In term neonates, the PBPK model was validated using individual plasma concentration–time data obtained from a study conducted at the University Hospital Leuven (UHL), Belgium. The methods, including the ethics approval (internal study number B‐32220084836) have been previously published,^39^ a summary of which is reported in Section S2 (Table S3). Patient consent was not required for this study.

#### Determination of Dose and Clearance Relationship

To determine the impact of PG dose on total clearance of PG, a dosing range which largely reflects the variation of doses which have been reported in literature was chosen. Therefore, the clearance of PG over a range of doses between 0.75 and 7500 mg/kg given intravenously as 6 hourly, 8 hourly, 12 hourly, and daily dosing were determined. This dosing range captures the recommended daily dosing of PG recommended by the EMA and FDA and reflects the potential daily dose of PG which may be administered through a wide range of intravenous or oral formulations. The lower dose range mirrored doses whereby linearity is expected in both adults and neonates, while the upper dose range reflected the doses which may be administered clinically but which may be expected to be associated with the saturation of PG clearance. The dose versus clearance comparison was performed in adults and neonates in all dosing scenarios. In this exercise, a simulated “observed” PG clearance, refers to the “dynamic” or “apparent” total clearance, which quantifies the impact of hepatic clearance and saturation as well as renal clearance capacity in both populations was used. This simulated “observed” clearance was calculated based on the simulated concentration–time profiles using the built‐in NCA tools within the Simcyp simulator and was equal to AUC/dose. Saturation of simulated “observed” clearance was determined by a 1% deviation from linearity (r^2^ = 0.98) using GraphPad prism (V9).

Finally, the concentration–time profile of PG at similar dosing conditions were determined to identify the dosing conditions associated PG with plasma levels associated with toxicity (PLAT). The PLAT of PG plasma level was set at 580 mg/L, based on Wilson et al (2005)^4^ and the model was used to identify a potential total daily dosing which may be suitable for adults and neonates.

#### Evaluation of Model Predictive Performance

To evaluate the predictive performance of the model, 2‐fold prediction of observed data and visual predictive check (VPC) approaches were employed. These methods of assessing the predictability of a model are generally accepted by PBPK modelers and drug regulatory bodies and have been employed successfully in similar studies.^18^, ^45^, ^46^ If observed and predicted area under the curve (AUC) and total systemic clearance (CL) were within 2‐fold in ∼90% of the study cases of comparison and if ∼90% of clinically observed studies had virtually all of their concentration versus time profile lying within the 5th and 95th percentiles of predicted concentration versus time profiles, the model predictability was deemed adequate.

## Results

### In Vitro Enzyme Kinetic Parameters of PG

The rate of NADH production with increasing PG concentration fitted the Michaelis–Menten model (Figure S2), and yielded K_m_ and V_max_ values of 25.1 mM and 1.57 nmole/min/mg of protein, respectively (Table 2) following curve fitting on GraphPad Prism. As for every 1 mole of NADH formed, 1 mole of PG is used up, the rate of NADH production equates the rate of PG metabolism in HLC (Figure S1).

**Table 2:** NADH Formation or PG Disappearance Kinetics Parameters in Human Liver Cytosol

**V_max_ (nmole/min/mg of protein)**	1.57 (1.41‐1.74)
**K_m_ (mM)**	25.1 (15.7‐39.2)

V_max_ represents maximum rate of metabolism while K_m_ represents the substrate concentration at which half the maximum rate of metabolism in achieved. Data in parentheses represents the 95% confidence interval.

### PG PBPK Model Performance in Adults and Term Neonates

**Figure 2:**
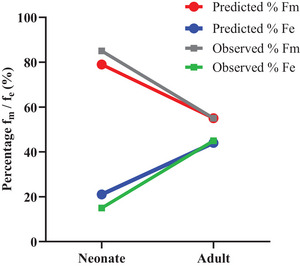
Mean contributions of hepatic metabolism and renal excretion to PG clearance in adults and neonates. Fm, fraction metabolized; Fe, fraction excreted.

The middle‐out approach of predicting PG with hepatic in vitro clearance and in vivo renal contribution lead to successful prediction of clearance contribution of PG in adults and neonates, with fraction metabolized and fraction excretion proportions similar to those published in the literature in the respective age groups. In both age groups, metabolism contributes mostly to the overall systemic clearance with a higher proportion of hepatic clearance in the two groups (85% observed and 79% predicted in neonates vs 55% observed and 55% predicted in adults) relative to renal clearance (15% observed and 21% predicted in neonates vs 45% observed and 45% predicted in adults) (Figure 2).^39^, ^43^, ^47^ Also, the PBPK model predicted the total clearance of PG of 8.53 and 0.083 L/h in adults and neonates, respectively, which were within 2‐fold has been reported in the literature (Table 3). Model validation involving specific published pharmacokinetic parameters on PG in adults and neonates is reported in Section S1.

**Table 3:** Predicted and Observed Clearances of PG in Adults and Neonates

	Adults		Neonates	
	Predicted	Observed	Predicted	Observed
Systemic CL (L/h)	8.5 (28)	7.5^#^ ^44^	0.083 (53)	0.085 (4.9)^38^
Hepatic CL (L/h)	4.6 (45)	4.2*^#^	0.063 (43)	0.056 (8.1)^42^
Renal CL (L/h)	3.7 (23)	3.3*^#^	0.011 (130)	0.0098 (11.8)^42^

Predicted values represent the geometric mean while the observed values represent the median estimates. All data in parenthesis are the percentage coefficient of variation (%CV).

^#^ %CV not reported; *estimated from a 55% versus 45% hepatic versus renal contribution to PG clearance in adults.^48^

Predicted values represent the geometric mean while the observed values represent the median estimates. All data in parentheses are the percentage coefficient of variation (%CV). #: %CV not reported, *: estimated from a 55% versus 45% hepatic versus renal contribution to PG clearance in adults.^47^

### PG Dose Versus Clearance Relationships Across Age Groups

Using a daily cumulative dosing range of 0.75 to 7500 mg/kg, applying standard dosing frequencies, PG (ADH) saturation occurred with an increase in frequency of dosing administration.

**Figure 3:**
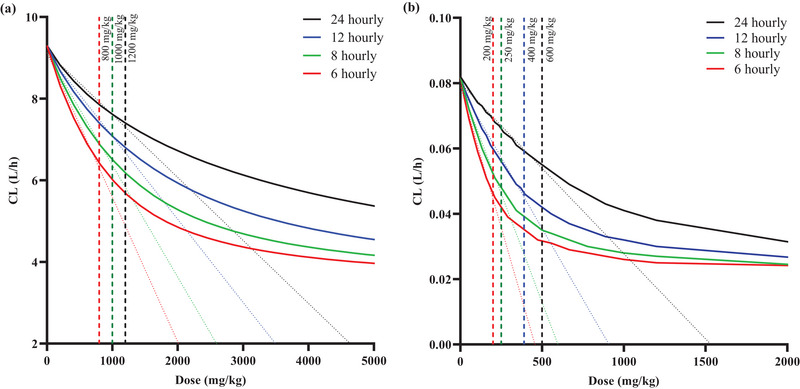
Dose (mg/kg per event) versus clearance relationship of PG in adults (a) and neonates (b). The maximum clearance of PG simulated over a range of PG doses simulated in adults (a) and neonates (b). The black, blue, green, and red solid lines represent the change in clearance with increasing PG doses following 24, 12, 8, and 6 hourly administrations, respectively. The corresponding colored diagonal lines represent the linear regression line of the linear phase of the dose versus clearance relationship. The black, blue, green, and red vertical dotted lines represent the dose at which the clearance was predicted to be saturated under 24, 12, 8, and 6 hourly dosing frequencies.

In neonates, saturation was predicted to occur when ≥200 mg/kg was administered 6 hourly (i.e., ≥ 800 mg/kg/day), but if dosed daily (every 24 h), saturation of PG clearance was expected when PG doses are greater than 600 mg/kg. However, in adults, ≥800 mg/kg PG dosed every 6 h (i.e., ≥ 3200 mg/kg/day) was predicted to result in saturation of PG clearance, but if dosed daily (every 24 h), saturation of PG clearance was expected at PG doses greater than 1200 mg/kg (Figure 3).

### PG Plasma Concentration Associated with PG Toxicity

**Figure 4:**
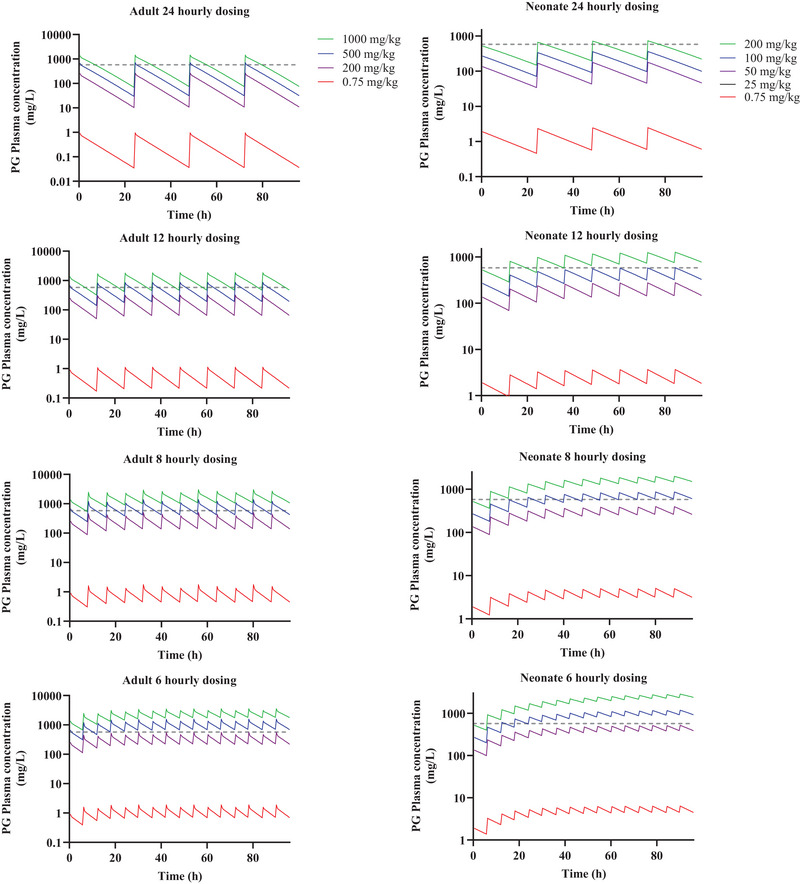
Predicted PG concentration–time profiles at standard dosing frequencies in adults (left panels) and neonate (right panels). In adults (left panels), the green, blue, purple, and red profiles represent the mean predicted concentration–time profiles of PG dose at 1000, 500, 200, and 0.75 mg/kg, respectively, dosed as 24, 12, 8, or 6 hourly. In neonates (right panels), the green, blue, purple, and red profiles represent the mean predicted concentration–time profiles of PG dose at 200, 100, 50, and 0.75 mg/kg, respectively, dosed as 24, 12, 8, or 6 hourly. The grey horizontal dotted line represents the PLAT of PG (580 mg/L) according to Wilson et al (2005).^4^

**Figure 5:**
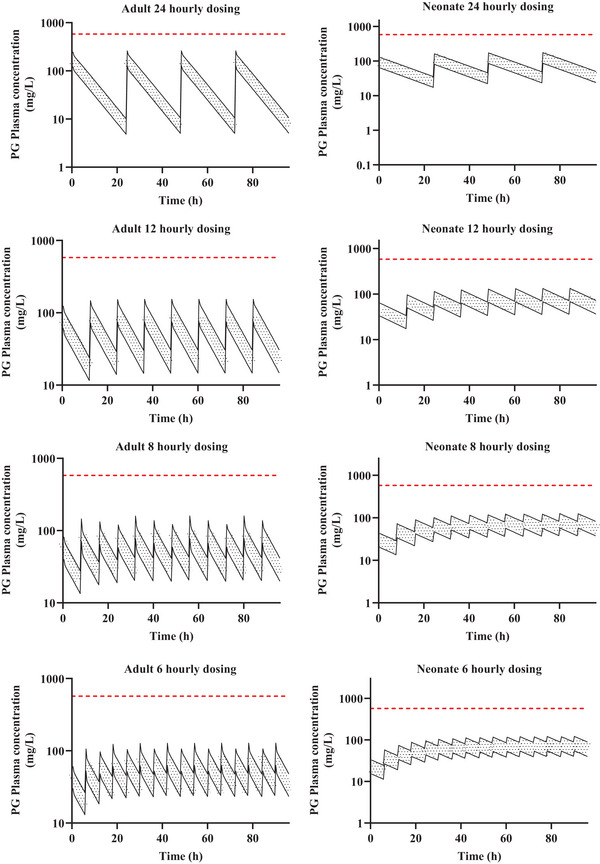
Plasma concentration–time profiles of proposed daily doses of PG in adults (left panels) and neonate (right panels) at standard dosing frequencies. In adults (left panels), the black line bordering the shaded area represents the concentration–time profiles following a total daily dose of 100‐200 mg/kg/day at standard dosing frequencies. In neonates (right panels), the black line bordering the shaded area represents the concentration–time profiles following a total daily dose of 25‐50 mg/kg/day at standard dosing frequencies. The red horizontal dotted line represents the PLAT of PG (580 mg/L) according to Wilson et al (2005).^4^

Predictions of plasma concentration versus time of PG in adults and neonate under standard dosing frequencies showed that in neonates, plasma levels of PG were below PLAT if 100 mg/kg PG was administered once daily, but PG plasma levels reach PLAT if the same dose was given 12, 8, and 6 hourly. Furthermore, when 50 mg/kg was administered at the standard dosing regimen, PG plasma levels were below PLAT at steady state though the 6‐hourly dosing schedule resulted in peak plasma levels close to PLAT. In adults, 200 mg/kg dosing on all standard dosing regimen resulted in plasma concentration below the PLAT, though the peak plasma level were close to PLAT on the 6‐hourly dosing schedules while at 500 mg/kg and in all standard dosing regimen, PG levels reached PLAT (Figure 4). A proposed total daily dose of 25‐50 mg/kg/day and 100‐200 mg/kg/day at standard dosing frequencies in neonates and adults, respectively, resulted in plasma concentration which was well below the PLAT (Figure 5).

## Discussion

Though PG has GRAS status, the toxic effects attributed to it ranging from hyperosmolality to multisystem organ dysfunction^47^, ^48^ warrant the need to better understand how disposition is linked to the toxic effects reported. Some attempts have been made to provide insight into this leading to PG toxicity being attributed to its accumulation and saturable clearance at high doses.^47^ Plasma concentrations above 580 mg/L (PLAT) have been associated with first signs of PG toxicity, such as increased osmolar gap.^4^

While these clearance and half‐life estimates provide some useful insights into the saturation potentials of PG clearance, the NTP's expert panel noted that those estimates were based on inconsistent inter patient clearance estimates at different dose conditions in a small subject cohort. Indeed, in the limited samples sizes (up to 16 subjects) in those studies, saturation was noted in one patient at about 7 g/day but occurred at higher doses in others. Also, they noted that above 12.6 g/day, there seemed to be a uniform reduction of clearance and the clearance of PG at doses below 5.1 g/day was not studied.^47^ The small sample size with extensive interindividual variability in PG PK parameters and the insufficient control of confounding factors which may have influenced the measured PK parameters of PG in these studies puts to question how closely these estimates align with the expected PG pharmacokinetics and potential toxicity.

Importantly, the clearance of PG in pediatrics is reduced due to immature ADH‐mediated clearance and renal function, making this subpopulation more vulnerable to toxicity due to accumulation and saturation.^39^ Therefore, and due to clinical observed toxicity, the acceptable daily intake of PG in children is reported to be about 50 mg/kg/day and 1 mg/kg/day in children older than 5 years and neonates, respectively, though it is not clear how the estimates of daily intake PG dose in these subpopulation was made.^6^ Thus, an approach which provides a mechanistic insight into the clearance of PG across a broader range of dosing scenario not only in adults but also in neonates was deemed necessary, and PBPK modeling and simulation was used to achieve this goal in this work.

In creating a PBPK model, the mechanistic data regarding compound physicochemical and pharmacokinetic properties of drugs are needed^17^ most of which were available publicly for PG (Table 1). However, as ADH‐mediated hepatic clearance attributes for 55% of total systemic clearance of PG, and this mechanism is subject to saturation^39^, the characterization of the saturation kinetics of PG clearance (Table 2) made it mechanistically possible to simulate PG clearance saturation.

This was the first study to determine the K_m_ of PG in class I ADH human liver homogenates. Morshed et al (1988) assessed the metabolic kinetics of PG in in vivo study experiments and found the K_m_ and V_max_ of PG in rats to be 17.86 mmole/kg and 8.33 mmole/kg/h, respectively^51^—these are equivalent to 21 mM and 1.88 nmole/min/mg of protein when scaled to in vitro rat liver cytosolic protein and are within 1‐fold of those determined in humans in this current study (25.1 mM and 1.57 nmole/min/mg of protein, respectively) (Table 2). The class 1 ADH metabolic activity of ethanol, having close structural properties to PG and metabolized via similar metabolic pathway, has been determined in human liver homogenates and the K_m_ is found to be 2.1mM^52^, ^53^ which is about 12‐fold lower than ADH‐mediated K_m_ of PG in human liver cytosol which was estimated in this study. This supports the notion that PG and ethanol are possibly involved in a competitive excipient–excipient interaction (EEIs) in pharmaceuticals containing them as both are cleared through similar metabolic pathways and ethanol has a lower K_m_ value for ADH activity compared to PG.^16^

This study focused on PG clearance in term neonates and adults due to data unavailability in older pediatric age groups. As ADH‐mediated hepatic clearance accounts for more than half of PG clearance in adults and this clearance mechanism is prone to saturation, incorporating the impact of enzyme ontogeny on the developmental clearance of PG in this model was important. Studies have reported the ontogeny of class 1 ADH enzymes including individual 1A, 1B, and 1C isozymes.^7^, ^54^ Bhatt et al (2017)^7^ reported neonatal abundance of individual ADH isoforms in comparison to adults and a combined relative ADH abundance of about 11% of adult level at birth whilst the total ADH activity in full term neonates was reported to be about 14% of adult levels.^8^ Other reports have suggested that neonatal ADH activity or expression is less than 20% of adult level.^40^, ^41^

With the full‐term ADH ontogeny incorporated in the PG model and accounting for immaturity in renal function, it was possible to predict the elimination profiles of PG in neonate (Figure 2 and Table 3). The in vitro determined ADH metabolic kinetics of PG resulted in predicted in vivo hepatic clearance (CL_H_) estimates which adequately reflected those reported in clinical trials in adults and after accounting for the impact of ontogeny on ADH activity in neonates, the resultant CL_H_ predicted were satisfactory (Figure 2, Table 3). More precisely, the median total systemic clearance CL_T_, CL_H_, and CL_R_ of PG predicted by the model (Table 3) were all less than 2‐fold of the observed in clinical studies in adults and neonates.^39^, ^43^, ^50^ The lower renal contribution to PG clearance observed in neonates in comparison to adults (Figure 2 and Table 3) suggests that though ADH‐mediated hepatic activity in comparison to renal function is higher in adults and neonate, the magnitude of hepatic activity in comparison to renal function is higher in neonate in comparison to adult. This is supported by the fact that the GFR in neonates is between 1% and 5% of adult GFR^55^ and compared to approximately 10%‐20% ADH activity in neonates in comparison to adults^8^, ^40^, ^41^ (Figure 2). The overall predictability of PG disposition in the adults and neonates was corroborated by good model performance in recovering actual observed concentration–time profiles, CL, and AUC parameters of PG in adults and in neonates (Figures S3 and S4 and Table S2).

A similar approach which employed PBPK modeling has been used to predict in vivo disposition for PG ether solvents—PG monomethyl ether (PGME) and PG monoethyl ether acetate (PMGEA) in rats and humans.^56^, ^57^ These solvents are used as commercial surface coatings and cleaners and despite their relatively more complex systemic clearance pathways in comparison to PG, the PBPK models employed in these studies, one of which incorporated the in vitro enzyme kinetics of the solvents, were able to acceptably predict their systemic exposure in rats and humans.^56^, ^57^

In adults, the PG model predicted that saturation of PG clearance is not expected to set in at doses greater 800, 1000, 1200, and 1200 mg/kg in 6, 8, 12, and 24 hourly respective dosing of PG, while in neonates PG clearance saturation was predicted to begin at above 200, 250, 400, and 600 mg/kg given 6, 8, 12, and 24 hourly, respectively (Figure 3). These estimates appear to differ from those reports in the EMA and USA NTP documents suggesting that PG is saturated at doses above 200 and 75 mg/kg/day, respectively, based upon studies conducted in adults in Speth et al (1987) and Yu et al (1985).^49^, ^50^ Furthermore, in adult and neonates, when considered as total daily dosing, the predicted PG dosing resulting in saturation correlated with PG PLAT in PG regimens of more than once daily but not in 24‐hourly regimen (Figure 4). This suggests that saturation of clearance has less association with toxicity at longer dosing interval, potentially because there is little superimposition on residual PG concentration following subsequent dosing. This finding supports the notion that the saturable clearance of PG may be linked to its PG toxicity as suggested in the EMA and NTP though the dosing estimates suggested by the EMA and NTP to be linked to toxicity are lower in comparison to what this current study shows.^6^, ^9^, ^47^ In a separate study, De Cock et al (2013),^39^ insinuated that in neonates, accumulation of PG in the plasma is likely due to immature hepatic or renal mechanisms and they did not attribute this to saturation of clearance mechanisms in neonates. This finding from this study suggests, therefore, that PG toxicity may occur before its clearance saturation is achieved and that accumulation may increase significantly once saturation is attained.

Studies have correlated osmolar gap of 8‐12 mOsm/L to plasma PG level up to 608 mg/L^12^, ^58^, ^59^ though a slightly more conservative estimate of 580 mg/L (PLAT) has been suggested to be associated with toxicity.^4^ These estimates are based on clinical observations in adults, not in neonates. The EMA proposed a 200 mg/kg/day dosing of PG to avoid levels associated with toxicity. Our simulations confirm that a total daily dosing below 200 mg/kg/day in adults is appropriate as the more the total daily dose deviates from this estimate, the more likely for PLAT (580 mg/L) to be achieved (Figures 4 and 5), therefore, we propose a total daily dose of 100‐200 mg/kg/day in any standard dosing frequency as suitable to avoid PLAT in adults (Figure 5). This is equivalent to about 250‐500 µg/kg/day of lorazepam in a 2 mg/mL lorazepam preparation containing 840 mg/mL of PG (Table S4). In neonates, accumulation of PG in the plasma occurred to greater extent (Figures 3 and 4). This reflects the immature elimination mechanisms, to result in a nearly 100‐fold lower clearance in neonates (Table 3). Similarly, in neonates, attainment of saturation depend on dosing frequency though when considered as a total daily dosing, the saturation thresholds for all dosing regimens were higher than the corresponding levels associated with toxicity (Figures 3 and 4). A total daily dose of 25‐50 mg/kg/day in any standard dosing frequency was therefore proposed to avoid PLAT in neonates (Figure 5). This is equivalent to about 60‐120 µg/kg/day of lorazepam in a 2mg/mL lorazepam preparation containing 840 mg/mL of PG (Table S4). Hyperosmolality and clinical symptom have been reported in infants at PG dose of 3000 mg/kg^60^, ^61^ a dosing level associated with elevated plasma concentrations due to underdeveloped clearance mechanisms and saturated clearance of PG in both adults and neonates according to our model prediction.

The challenge with developing the model is mainly around the sparsity of clinical data to validate the model's PK predictive performance across a broader range of doses and age groups. For example, though the ontogeny of ADH activity or expression across age groups has been reported,^7^, ^8^, ^54^ clinically observed concentration versus time profiles of PG across all age groups are not available publicly. Consequently, the impact of ADH ontogeny on PG clearance across pediatric age bands has not been demonstrated—though a study suggested based on clinically observed PG concentration data in four children—that PG hepatic and renal clearance both reach adult levels at about 5‐12 year olds.^62^ Therefore, the lack of robust PG concentration–time data in individuals across all age groups who have used a wide range of PG doses meant that model validation of PG PK was based on data for a limited dose range in neonates and adults.

De Cock et al (2014) highlighted the potential for inducible renal clearance of PG follow the first dose of PG administration in neonates,^43^ but this was not accounted for in the current study. Furthermore, it is important to mention that we assumed that only developmental pharmacokinetics explains the developmental toxicity associated with PG exposure in neonates. At present, we are unaware of any pharmacodynamic data on developmental toxicity, though there is a report on the apoptotic potential of PG in the developing central nervous system in a mouse model.^63^ This highlights the importance of characterizing CNS exposure in humans. In addition, certain PG toxicity have been attributed to EEI involving ethanol, for example, in the case with PG toxicity observed in neonate administered Kaletra was attributed to PG and ethanol competion for the same ADH‐mediated clearance pathway.^16^ This suggest that there is likely less tolerability for PG doses in formulations containing ethanol. The current PG study has not accounted for the impact of this potential EEI on PG exposure. Finally, these modeling efforts have not explicitly incorporated the impact of additional excipients which may impact the pharmacokinetics of PG more generally. Despite these limitations, the modeling efforts in this current study have provided reasonable estimates of the relationship between PG dose, its linear and zero‐order clearance kinetics and its toxicity over a larger dosing range in both adults and neonates from a mechanistic viewpoint.

## Conclusion

In conclusion, our combination of in vitro and mechanistic modeling techniques in determining the clinical implications of saturability of PG clearance in adults and neonates confirmed that PG clearance saturation and toxicity is possible at doses which have been used clinically and reported in the published literature. We have also demonstrated that the PG doses resulting in clinical toxicity are lower than those associated with its clearance saturations. Finally, we propose a total daily dose of 100‐200 mg/kg/day in adults and 25‐50 mg/kg/day in neonates at any standard dosing frequency to avoid toxicity or saturation of clearance in pharmaceutical formulations which do not contain excipients that PG may interact with and in patients without impaired clearance of PG.

## Conflicts of Interest

The authors declare no conflicts of interest.

## Funding

The authors did not receive financial support from any organization for the submitted work. No funding was received to assist with the preparation of this manuscript. No funding was received for conducting this study. No funds, grants, or other support was received.

## Supporting information



Supporting Information

## Data Availability

The data that support the findings of this study are available from the corresponding author upon reasonable request.
