# Supplementary material for: Exploring the Impact of Developmental Clearance Saturation on Propylene Glycol Exposure in Adults and Term Neonates Using Physiologically Based Pharmacokinetic Modeling
Source: J Clin Pharmacol. 2024 Oct 15;65(3):272–84. doi: 10.1002/jcph.6150 (PMC11867916; doi:10.1002/jcph.6150)
Supplement: Supplementary file 1 — Supporting Information [file JCPH-65-272-s001.docx]

**SUPPLEMENTARY INFORMATION**

**SECTION 1: Supplementary methods and results**

**Supplementary Methods**

**NADH standard curve determination**

A standard NADH curve (Figure S6 – supplementary material) was determined by measuring the absorbance of NADH at 1 µM, 3 µM, 10 µM, 30 µM, 100 µM concentrations. The absorbance of NADH was taken at wavelength of 340 nm using the Spectramax M2e multi-detection microplate reader.

**Optimisation of cytosolic concentrations and incubation time for metabolic kinetic experiments**

To determine the cytosolic concentration and incubation time with a linear metabolism of PG, protein concentrations of 0.1, 0.25, 0.5, 0.75 and 1 mg/ml were incubated in 5mM PG, 0.1 M phosphate buffer (KH2PO4 / K2HPO4, pH 7.4) and 1 mM NAD+. The absorbance of NADH was taken at wavelength of 340nm using the Spectramax M2e multi-detection microplate reader and the absorbance readings were taken at 0, 5, 10, 15, 20, 25, 30 and 45 minutes at an incubation temperature of 37^o^C. The optimal cytosolic protein concentration and incubation time for linear metabolism of PG was determined to be 0.5 mg/ml and 20 minutes respectively.


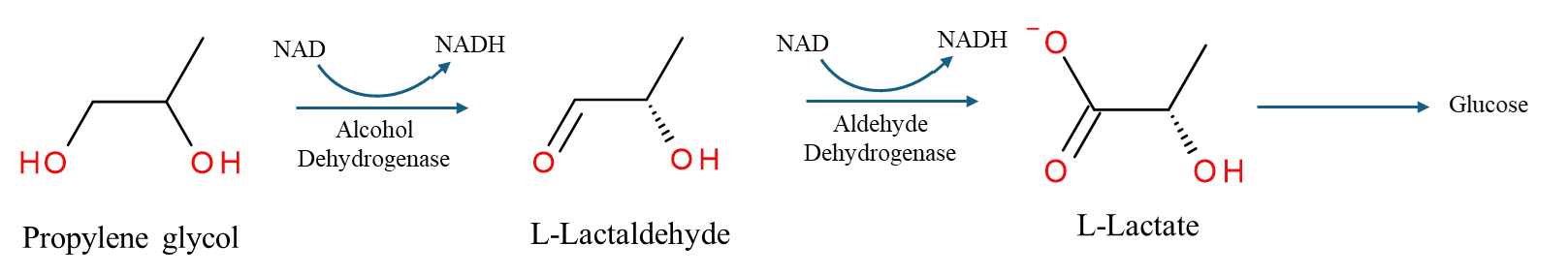


**Figure S1: ADH mediated chemical reaction of PG**

**Table S1: ADH enzyme expression in neonates and adults**

| **Enzyme isoform** | **ADH1A** | **ADH1B** | **ADH1C** | **Total** |
| --- | --- | --- | --- | --- |
| Neonates* (pmol/mg of cytosolic protein) | 158 | 658 | 70 | 886 |
| Adult (pmol/mg of cytosolic protein) | 426 ^1^ | 5220 ^2^ | 2598 ^3^ | 8244 |
| Percentage of adult expression levels % | 37.0 ^4^ | 12.6 ^4^ | 2.7 ^4^ | 10.6 |

***:** *Calculated based on the actual expression levels in adult ^1^ and the estimated percentage of adult expression ^4^*

**Supplementary results**

The relationship between the rate of NADH production and increasing concentration of PG in human liver cytosol is shown in Figure 3 and the derived kinetic parameters (V_max_ and K_m_) are shown in Table 2. Between 1 and 50 mM PG, the rate NADH production increased linearly, after which the rate of NADH approached saturation. The rate of NADH production with increasing PG concentration fitted the Michaelis Menten model (Figure 3).

**Figure S2: NADH formation rate in the presence of PG in human liver cytosol.**

Data points represent average of triplicate rates of NADH formation measured for each PG substrate concentration and the error bar represent the standard error of mean (SEM). The derived kinetic constants of PG disappearance are presented in Table 2. The rate or reaction and PG concentration data points were fitted to the Michaelis-Menten equation.

### PG PBPK model validation in adults

In adults, the PG model predicted AUCs and CL in adults which were within 2-folds of the AUC and CL values reported in the clinical studies used for model validation with the exception of the ratio between observed and predicted CL in the 20700 mg PG Speth et al (1987) study ^5^ (Table S1). Despite this, the individual and mean concentration versus time data points in the Speth et al (1987) ^5^ and Yu et al (1985) ^6^ studies respectively, were within the 5^th^ and 95^th^ percentile of predicted concentration versus time data points (Figure S1). Since these were only two studies in adult where the plasma concentration time profile for PG were reported and the reported profiles involved limited subjects size, the reported profiles in these studies may not have captured variability in plasma concentrations sufficiently and this may explain the discrepancy in CL prediction in the 20700 mg PG Speth et al (1987) study ^7^ (Table S2).

**Figure S3: Simulated plasma concentration–time profile of PG in adults.**

Clinical studies used for validation involved oral and intravenous administration of PG. Details of dosing regimen are reported in supplementary material Table S1. Open red circles in a) and b) Yu et al (1985); c) and d) Speth et al (1987); represent observed individual concentration-time points while open blue square in a) and b) represents the mean of concentration-time points retrieved from Yu et al (1985) clinical studies. Solid black lines represent population mean predictions, dashed lines represent 5^th^ and 95^th^ percentiles of prediction and shaded grey area represents predicted concentrations-time profiles within the 5^th^ and 95^th^ percentile.

**Table S2: Predicted and observed pharmacokinetic parameters of PG in adults and neonates**

| **Population** | **Clinical study** | **Predicted** | | | **Observed** | | | | **Ratio** | |
| --- | --- | --- | --- | --- | --- | --- | --- | --- | --- | --- |
|  |  | **AUC (SD)(mg.h/ml)** | **CL (SD) L/h/kg** | | **AUC (SD) (mg.h/ml)** | | **CL (SD) L/h/kg** | | **AUCR** | **CLR** |
| **Adult** | Speth et al (a) | 1.67 (0.44) | | 0.116 (0.033) | | 1.62* | | 0.33* | 1.0 | 2.8 |
|  | Speth et al (b) | 3.50 (0.91) | | 0.113 (0.032) | | 6.43* | | 0.21* | 1.8 | 1.9 |
|  | Yu et al (c) | 2.58 (0.73) | | 0.117 (0.035) | | 3.23 (0.95) | | 0.106 (0.023) | 1.3 | 0.9 |
|  | Yu et al (d) | 5.40 (1.42) | | 0.111 (0.032) | | 7.74 (2.43) | | 0.079 (0.015) | 1.4 | 0.7 |
| **Full term neonate** | De Cock et al (e) | 0.73 (0.34) | | 0.028 (0.015) | | NA | | NA | - | - |
|  | De Cock et al (f) | 0.84 (0.39) | | 0.028 (0.015) | | NA | | NA | - | - |
|  | De Cock et al (g) | 2.63 (1.24) | | 0.028 (0.015) | | NA | | NA | - | - |
|  | De Cock et al (h) | 1.10 (0.52) | | 0.028 (0.015) | | NA | | NA | - | - |
|  | De Cock et al (i) | 0.64 (0.32) | | 0.028 (0.015) | | NA | | NA | - | - |
|  | De Cock et al (j) | 0.74 (0.37) | | 0.028 (0.015) | | NA | | NA | - | - |
|  | De Cock et al (k) | 1.87 (0.93) | | 0.028 (0.015) | | NA | | NA | - | - |
|  | De Cock et al (l) | 0.56 (0.28) | | 0.028 (0.015) | | NA | | NA | - | - |

AUC: area under the concentration versus time curve; CL: total systemic clearance; SD: standard deviation; AUCR: AUC ratio; CLR: CL ratio; AUCR and CLR were calculated by dividing the predicted mean values from each study by the respective mean observed studies. *: Data was for individual subject; therefore, no SD is available. a-s: study labels as seen in the Figures S1- S2. Summary of clinical studies used for validation in the supplementary material Table S2.; NA: not available.

### PG model validation in neonates

In neonates, PG concentration versus time data with details of dosing regimen is scarce in literature and as the time of conducting this current study, there were no publicly available PG plasma concentration time profiles which could be used to validate the paediatric PG model except those obtained from UHL ^8^ The paediatric PG model sufficiently recovered the clinically observed concentration versus time profiles reported in neonates based on the De Cock et al (2013) study as the spread of observed concentration-time data points mostly fell within the 5^th^ and 95^th^ percentile of predicted concentration-time profiles under different dosing situations (Figure S2). A ratio between the predicted and observed AUC and CL parameters were not determined as the observed AUC and CL parameters were not available in the neonates used for validation.

**Figure S4:** **Simulated plasma concentration–time profile of PG in full term neonates and children.**

Clinical studies used for validation involved intravenous administration of PG. Details of dosing regimen implemented are reported in supplementary material Table S1. Open red circles in d) and k) represent observed individual concentration-time points retrieved from De Cock et al (2013) clinical studies. Solid black lines represent population mean predictions, dashed lines represent 5^th^ and 95^th^ percentiles of prediction and shaded grey area represents predicted concentrations-time profiles within the 5^th^ and 95^th^ percentile.

**SECTION 2: Supplementary figures and tables**


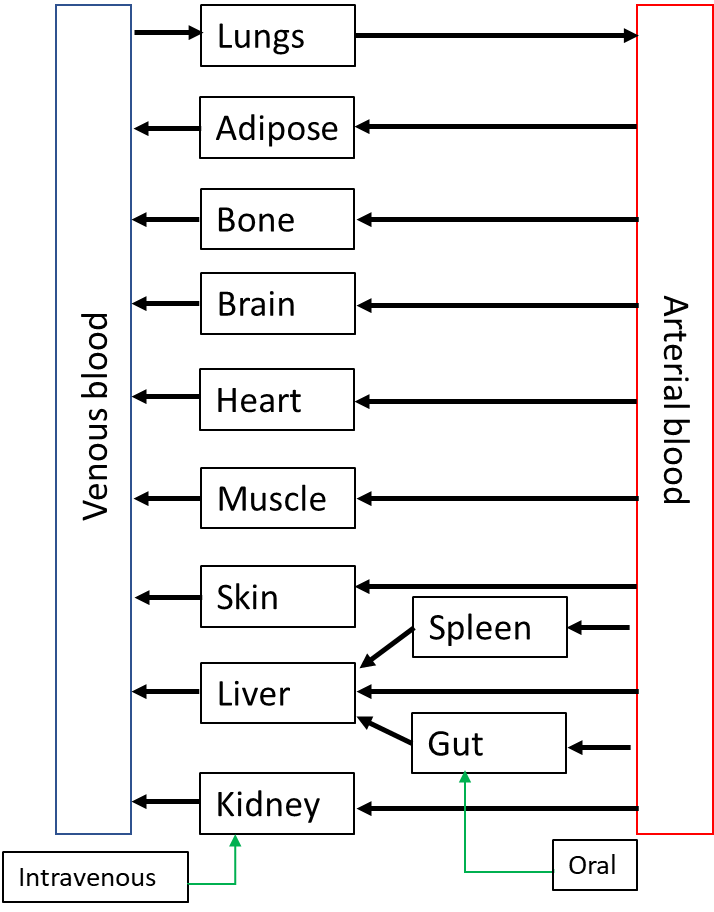


**Figure S5: The whole-body PBPK model structure used for PG model development. Adapted from Sadighi et al (2021) ^9^.** Black arrows represent direction of blood flows; green arrow represent dose administration.

| **Population** | **Clinical study** | **Dose** | **Frequency** | **Administration type** | **No of subjects** |
| --- | --- | --- | --- | --- | --- |
| **Adults** | Speth et al. ^5^ | 7.5 g/m^2^ | Once | Infusion | 1 |
|  |  | 15 g/m^2^ | Once | Infusion | 1 |
|  | Yu et al ^6^ | 20.7 g | 8 hourly | Oral | 2 |
|  |  | 41.4 g | 12 hourly | Oral | 2 |
| **Neonate** | De Cock et al ^8^ | 56 mg | Once | Infusion over 15 minutes | 3 |
|  |  | 64 mg | Once | Infusion over 15 minutes | 1 |
|  |  | 84 mg | Once | Infusion over 15 minutes | 1 |
|  |  | 196 mg | Once | Infusion over 15 minutes | 2 |
|  |  | 43 stat, then 22 mg | 6 hourly | Infusion over 15 minutes | 1 |
|  |  | 48 stat, then 24 mg | 6 hourly | Infusion over 15 minutes | 4 |
|  |  | 56 stat, then 28 mg | 6 hourly | Infusion over 15 minutes | 2 |
|  |  | 140 stat, then 35 mg | 12 hourly | Infusion over 20 minutes | 1 |

**Table S3: Summary of clinical data using for model validation**

**Figure S6: NADH standard curve**

| **Product name** | **Formulation type** | **API per ml** | **Recommended daily dose of API (mg/kg/day)** | **PG in formulation (mg/ml)** | **Amount of PG administered daily (mg/kg/day)** | |
| --- | --- | --- | --- | --- | --- | --- |
| Lorazepam | IV | 2 | 0.2 | 840 | 84 | |
| Diazepam ^a^ | IV | 5 | 0.6 - 0.8 | 550 | 66 - 88 | |
| Phenytoin ^a^ | IV | 50 | 5 - 10^#^ | 414 | 41.4 - 82.8 | |
| Phenobarbital ^a, b^ | IV | 60 | 20 | 936 | 312 | |
| Digoxin ^a^ | IV | 0.25 | 0.001 - 0.035 | 425 | 1.7 - 59.5 | |
| Paracetamol ^b^ | oral suspension | 24 | 60 | 2.9 | 7.25 | |
| Amiloride ^b^ | oral solution | 1 | 0.1 - 0.2 | 20.7 | 2.1 - 4.1 | |
| Propranolol ^a, b^ | oral solution | 2 | 0.25 - 0.5 | 20 | 5 | |
| Furosemide ^b^ | oral solution | 4 | 0.5 - 2 | 0.07 | 0.009 - 0.04 | |
| *a: may contain ethanol; b: other strengths available in formulation type. API: active pharmaceutical ingredient; IV: intravenous.* *#: dose excludes starting dose; *: summary should be interpreted cautiously because sometimes, different formulations are marketed (containing different concentrations of the API and excipients), and the information on concentrations of excipient may be difficult to access. Data on formulation type, API per ml and PG content per ml retrieved from electronic medicine compendium ^10^; data on maximum daily dose of API retrieved from British National Formulary for Children ^11^.* | | | | | |  |

**Table S4: Examples of formulations of drugs used in term neonates containing propylene glycol***

**References:**

1. Di L, Balesano A, Jordan S, et al. The Role of Alcohol Dehydrogenase in Drug Metabolism: Beyond Ethanol Oxidation. AAPS J. 2021;23(1):20.

2. Bhatt DK, Gaedigk A, Pearce RE, et al. Age-dependent Protein Abundance of Cytosolic Alcohol and Aldehyde Dehydrogenases in Human Liver. Drug Metab Dispos. 2017;45(9):1044-1048.

3. Berezhkovskiy LM. Volume of distribution at steady state for a linear pharmacokinetic system with peripheral elimination. J Pharm Sci. 2004;93(6):1628-1640.

4. van Groen BD, Nicolai J, Kuik AC, et al. Ontogeny of Hepatic Transporters and Drug-Metabolizing Enzymes in Humans and in Nonclinical Species. Pharmacol Rev. 2021;73(2):597-678.

5. Speth PAJ, Vree TB, Neilen NFM, et al. Propylene-Glycol Pharmacokinetics and Effects after Intravenous-Infusion in Humans. Therapeutic Drug Monitoring. 1987;9(3):255-258.

6. Yu DK, Elmquist WF, Sawchuk RJ. Pharmacokinetics of propylene glycol in humans during multiple dosing regimens. J Pharm Sci. 1985;74(8):876-879.

7. Giacoia GP, Miranda R, West KI. Measured vs calculated plasma osmolality in infants with very low birth weights. Am J Dis Child. 1992;146(6):712-717.

8. De Cock RF, Knibbe CA, Kulo A, et al. Developmental pharmacokinetics of propylene glycol in preterm and term neonates. Br J Clin Pharmacol. 2013;75(1):162-171.

9. Sadighi A, Leggio L, Akhlaghi F. Development of a Physiologically Based Pharmacokinetic Model for Prediction of Ethanol Concentration-Time Profile in Different Organs. Alcohol Alcohol. 2021;56(4):401-414.

10. Electronic medicine compedium Available: <https://www.medicines.org.uk/emc>. Accessed 09 September, 2024.

11. National Institute for Health and Care Excellence (NICE). British National Formulary for Children (BNFC) Available: <https://bnf.nice.org.uk/>. Accessed 09 September, 2024.
